# Wnt Signaling Is Regulated by Endoplasmic Reticulum Retention

**DOI:** 10.1371/journal.pone.0006191

**Published:** 2009-07-10

**Authors:** J. Susie Zoltewicz, Amir M. Ashique, Youngshik Choe, Gena Lee, Stacy Taylor, Khanhky Phamluong, Mark Solloway, Andrew S. Peterson

**Affiliations:** Ernest Gallo Clinic & Research Center, Emeryville, California, United States of America; University College London, United Kingdom

## Abstract

Precise regulation of Wnt signaling is important in many contexts, as in development of the vertebrate forebrain, where excessive or ectopic Wnt signaling leads to severe brain defects. Mutation of the widely expressed *oto* gene causes loss of the anterior forebrain during mouse embryogenesis. Here we report that *oto* is the mouse ortholog of the gpi deacylase gene *pgap1*, and that the endoplasmic reticulum (ER)-resident Oto protein has a novel and deacylase-independent function during Wnt maturation. Oto increases the hydrophobicities of Wnt3a and Wnt1 by promoting the addition of glycophosphatidylinositol (gpi)-like anchors to these Wnts, which results in their retention in the ER. We also report that *oto*-deficient embryos exhibit prematurely robust Wnt activity in the Wnt1 domain of the early neural plate. We examine the effect of low *oto* expression on Wnt1 *in vitro* by knocking down endogenous *oto* expression in 293 and M14 melanoma cells using shRNA. Knockdown of *oto* results in increased Wnt1 secretion which is correlated with greatly enhanced canonical Wnt activity. These data indicate that *oto* deficiency increases Wnt signaling *in vivo* and *in vitro*. Finally, we address the mechanism of Oto-mediated Wnt retention under *oto*-abundant conditions, by cotransfecting Wnt1 with gpi-specific phospholipase D (GPI-PLD). The presence of GPI-PLD in the secretory pathway results in increased secretion of soluble Wnt1, suggesting that the gpi-like anchor lipids on Wnt1 mediate its retention in the ER. These data now provide a mechanistic framework for understanding the forebrain defects in *oto* mice, and support a role for Oto-mediated Wnt regulation during early brain development. Our work highlights a critical role for ER retention in regulating Wnt signaling in the mouse embryo, and gives insight into the notoriously inefficient secretion of Wnts.

## Introduction

Wnts are a family of secreted, cysteine-rich signaling proteins that affect cell fate and proliferation in a wide variety of contexts [1], [2]. Because vertebrate Wnt proteins are highly conserved and more hydrophobic than predicted based on sequence, it has been difficult to generate antisera that recognize Wnts *in vivo*, or to visualize them at endogenous expression levels [3], [4], [5], [6], [7], [8]. Wnts are also inefficiently secreted, tending to linger in the endoplasmic reticulum (ER) [1]. These challenges have delayed understanding of the distribution and biochemical nature of Wnts in vertebrate organisms. Although a great deal is known about the molecular pathways that mediate the Wnt response, little is known about the mechanisms underlying Wnt production [8], [9], [10]. In the latter regard, recent advances have revealed that Wnts are lipid-modified in Wnt-producing cells *in vivo*, by the O-acyltransferase *porcupine*. Palmitic and palmitoleic acids are covalently linked to Wg, Wnt1 and Wnt3a during their maturation, which serve to stimulate their secretion and signaling activities [5], [7], [11], [12], [13]. Wg also associates with lipoprotein particles, which allows signaling over many cell diameters [7], [14]. These recent insights indicate that Wnt production and activity rely on hydrophobic mechanisms, and further suggest that Wnt function is integrally associated with the lipid environment of cells [9], [10].

Evidence from Drosophila, *C. elegans*, *Xenopus*, zebrafish, chicken, and mouse has indicated that Wnts act as morphogens in the developing embryo, forming concentration gradients across cell fields which generate pattern [14], [15], [16], [17], [18], [19], [20], [21], [22]. Wnts play an important role in the generation of antero-posterior (AP) pattern along the developing neural axis [22]. Evidence indicates that early neural plate cells are transformed into more posterior fates by a gradient of Wnt activity that is lowest at the anterior end of the neuraxis, and highest posteriorly [16], [17], [18], [19], [23], [24]. Because the forebrain forms at the low end of the Wnt gradient, it is particularly and exquisitely sensitive to the posteriorizing influences of Wnt signals [25], [26], [27], [28], [29]. Forebrain formation will not occur unless Wnt activity in the anteriormost neurectoderm is severely limited [18], [23], [30], [31], [32].

The recessive, lethal *oto^xray^* mutation causes forebrain truncations in mice that closely resemble those in *dkk1*, *ICAT*, *six3* and *hesx1* mutants [23], [33], [34], [35], [36], [37], [38]. Dkk1 and ICAT are extracellular and intracellular Wnt inhibitors, respectively. Dkk1 is secreted by the prechordal plate mesendoderm and protects forebrain development in apposed neurectoderm [39], [40], [41], [42]. The *six3* and *hesx1* transcription factors are expressed in prechordal neurectoderm from early neural plate stages, and both are repressed by Wnt signaling [23], [30], [37], [43], [44]. Six3 in turn directly represses transcription of Wnt1, which is normally expressed in immediately posterior medial neural plate cells [23], [45], [46]. Hesx1 may indirectly repress Wnt1 signaling [30]. These repressive interactions serve to restrain Wnt1 to its medial domain, in order to generate and maintain an anterior zero-to-very low Wnt activity zone, which is required for the development of forebrain fates. Ectopic Wnt1 expression and Wnt activity in the anteriormost neural plates of *six3* and *hesx1*-deficient embryos are reported as the molecular bases of the failure of forebrain development in these mice [23], [30]. Precise regulation of Wnt signaling is thus critical for normal forebrain development, especially at early stages, when the neurectoderm is fate-labile [47].

We have used a forward genetics approach in our study of the *oto* mouse [33]. To add to our previous phenotypic characterization of *oto*, we now reveal the identity of the *oto* gene, and present an investigation into the molecular function of the Oto protein. Phenotypic parallels with Wnt-inhibitor mutant mice initially suggested that *oto* might act as a Wnt antagonist during early brain development. Instead, we find that Oto regulates Wnt secretion via a novel mechanism. We show that Oto is a widely expressed, ER-resident glycoprotein involved in adding atypical glycophosphatidylinositol (gpi) anchors to Wnts 1 and 3a, which results in their retention in the ER. We further show that Oto is required for the correct initiation of Wnt signaling in the Wnt1 domain of the early neural plate. In *oto*-deficient embryos, neurectodermal Wnt signaling in the Wnt1 domain begins prematurely and robustly, which is consistent with the loss of Oto-mediated Wnt retention. The early neural plate in *oto* mutants thus exhibits an abnormally large medial domain of Wnt activity, and the *oto* embryo subsequently develops with a truncated forebrain.

Our results reveal the existence of a novel Oto-dependent mechanism that retains Wnts in the ER of Wnt producing cells. We also present evidence that intracellular cleavage of gpi anchor lipids stimulates Wnt secretion. We propose that gpi-anchoring of Wnts provides a means of accumulating, and then releasing, a regulated burst of Wnt ligands. Conversely, in the absence of *oto*, we show that active Wnt flows out of the cell in a relatively uninhibited manner. We thus describe a new mechanism involved in the production of Wnt ligands, that has an important role during mammalian embryogeneis. Given the widespread expression of *oto* throughout mammalian life, this novel mode of Wnt regulation likely extends to other Wnt-dependent processes in development and disease.

## Results

### Identity of the oto gene

**Figure 1:**
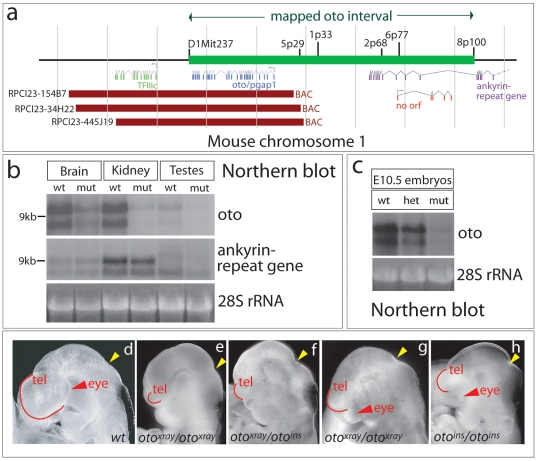
Identification of the *oto* gene. a.Genes in the vicinity of the *oto* interval (indicated by the green bar, with mapping markers above) are shown. Red bars represent rescuing BACs. b. Gene expression in wildtype (wt) and an extremely rare adult *oto* mutant (mut) shows reduced *oto* mRNA in mutant tissues. c. *oto* expression is reduced in E10.5 *oto* heterozygotes (het) and homozygotes (mut). d–h. Heads of E9.5−10 embryos (side views, anterior left), showing telencephalic vesicle (red arc), eye if present (red arrowhead), and isthmus (yellow arrowhead). Both *oto* alleles yield similar severely (e, f) and moderately (g, h) affected mutants.

The recessive, lethal *oto^xray^* mutation was mapped to a 284 kilobase interval on chromosome 1 which contains two genes, *TFIIIc* and *oto/pgap1* (Fig. 1a). Northern blot analysis reveals reduced expression of only *oto/pgap1* in mutant mouse adult tissues and *oto*-deficient embryos (Fig. 1b,c). The mRNA size and cDNA sequence of this gene is normal in mutants, indicating that the x-ray lesion disrupts a regulatory region which attenuates transcription. To test whether this hypomorphic allele is responsible for the *oto^xray^* mutant phenotype, we used a BAC (bacterial artificial chromosome) transgenic rescue approach. Three BACs, each containing the complete *oto/pgap1* gene, individually rescued the developmental defects and restored viability to *oto* homozygotes (Supplementary Table S1). The generation of a second insertional allele (*oto^ins^*, Supplementary Fig. S1) provides further evidence that the correct gene was identified [48]. Doubly heterozygous *oto^xray^/oto^ins^* and *oto^ins^/oto^ins^* embryos show variable forebrain defects identical to those in *oto^xray^/oto^xray^* embryos (Fig. 1d–h). The *oto^ins^/oto^ins^* mutant in Fig. 1h was not the most severe *oto^ins^/oto^ins^* mutant recovered; homozygous *oto^ins^* mutants can be as severely affected in the forebrain as *oto^xray^* mutants.

### Oto increases Wnt hydrophobicity

**Figure 2:**
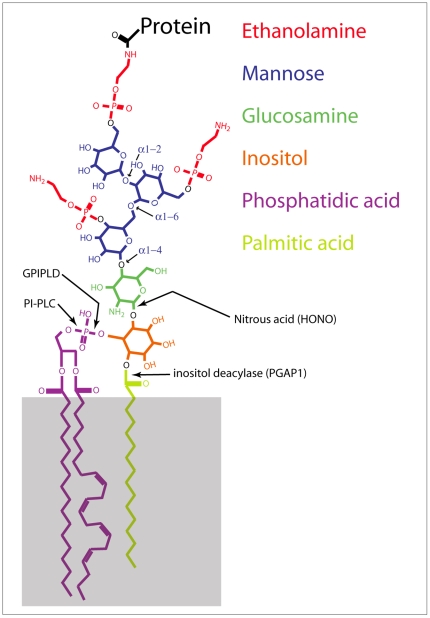
Diagram of a gpi anchor. Individual components are color-coded as shown. Gray box represents the outer leaflet of the cell membrane. Sites of cleavage by PI-PLC, nitrous acid (HONO), GPI-PLD, and inositol deacylase are indicated.

**Figure 3:**
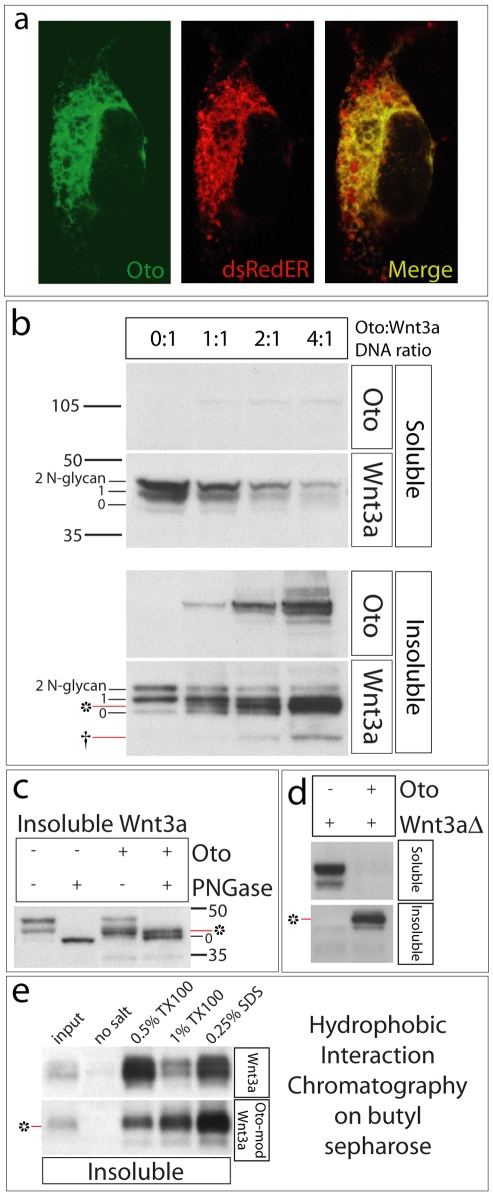
Oto localizes to the ER and increases Wnt hydrophobicity. a. Gfp-Oto (transmembrane) and the ER marker dsRedER (luminal) were co-expressed and visualized in a single cell. They overlap significantly (merge, yellow), illustrating that Oto resides in the ER like its rat ortholog PGAP1. Western blots from transfected 293 cells are shown in b–e. * = Oto-modified Wnt3a, † = degradation product. 0–2 indicates the number of N-glycans on Wnt3a. b. Co-expression with Oto converts Wnt3a to an insoluble isoform migrating at the novel position of 42 kDa (*). c. PNGaseF-mediated deglycosylation of Oto-modified Wnt3a does not change its migration. d. Wnt3a lacking amino acids 31–107 cannot be palmitoylated and therefore is soluble. When co-expressed with Oto, it becomes insoluble. e. Most Wnt3a elutes with the non-ionic detergent TX100, while most Oto-modified Wnt3a elutes with SDS due to its increased hydrophobicity.

Oto is orthologous to rat PGAP1, an ER-resident transmembrane glycoprotein [38], [49]. PGAP1 has gpi deacylase activity; it removes palmitate from the inositol ring of gpi anchors [49] (Fig. 2). Because *oto* forebrain defects resemble those in Wnt inhibitor mutants [23], [30], [34], [35], [36], [37] and PGAP1 resides in the ER [49], we surmised that Oto might act on Wnts in the early secretory pathway. To explore this, we performed co-expression experiments in 293 cells. We chose Wnt3a initially because overexpression of *Xwnt3a* suppresses expression of anterior neural markers in neuralized animal caps [21], and because *wnt3a* mutant mice show *oto*-like vertebral transformations [50]. For detection purposes, Oto was amino-terminally tagged with a 3xflag epitope; Wnt3a was detected via a carboxy-terminal HA tag. Like PGAP1, Oto localizes to the ER in transfected cells (Fig. 3a). Oto is fully de-N-glycosylated with Endoglycosidase H (EndoH, not shown), an enzyme that cleaves immature, high mannose N-glycans from the protein core (NEB). Resistance to EndoH is conferred after a protein exits the ER, during processing in later secretory compartments [51]. The EndoH-sensitivity of Oto also indicates that Oto resides in the ER.

When expressed alone, Wnt3a protein appears in both RIPA-soluble and insoluble (pelleted) phases (Fig. 3b). Three bands appear in each phase; lowest is naked polypeptide, and middle and upper bands are singly and doubly N-glycosylated isoforms, respectively. In transient transfections, when the amount of Oto DNA is varied and Wnt3a DNA is constant, an interesting pattern emerges. When Oto is present, a new insoluble isoform of Wnt3a appears, at the expense of soluble Wnt3a. As Oto expression increases, more Wnt3a is converted to the hydrophobic isoform. This isoform is upshifted relative to naked Wnt3a, migrating at 42 kDa; smaller, insoluble degradation products are also observed. Since its formation is Oto-dependent, we will call the 42 kDa isoform Oto-modified Wnt3a. We use this designation for convenience and do not intend to imply that Oto directly catalyzes a modification reaction. After de-N-glycosylation with PNGaseF, Oto-modified Wnt3a still migrates at 42 kDa while Wnt3a expressed alone collapses to the 0 glycosylation state (Fig. 3c), revealing that the Oto-modification is not an N-glycan. Because the 4∶1 ratio of Oto:Wnt DNA yielded the most Oto-modified Wnt3a, this ratio was used in subsequent experiments, with the control being a 4∶1 DNA ratio of empty vector:Wnt or gfp:Wnt.

Hydrophobicity of Wnt3a can be increased by palmitoylation on a conserved cysteine, C77 [5], and by addition of palmitoleic acid to serine 209 (S209) [13]. Both of these lipidations are mediated by porcupine [12]. To determine whether Oto's modification of Wnt3a involves C77, we tested a Wnt3a deletion construct lacking the palmitoylated residue (Wnt3aΔ31–107). Wnt3aΔ31–107 is fully soluble in RIPA when expressed alone, but is completely shifted to the insoluble pellet when expressed with Oto (Fig. 3d). Thus the Oto modification appears to be independent of C77 palmitoylation. We did not directly address the possible role of S209 in this study.

To measure the apparently novel hydrophobicity change in Wnt3a caused by Oto co-expression, we performed hydrophobic interaction chromatography (HIC) using butyl sepharose. Proteins bind to butyl sepharose via their hydrophobic surfaces and moieties [7]. A protein's hydrophobicity can be measured by the stringency of its elution conditions [7]. Insoluble material only, isolated from cells expressing full length Wnt3a or Oto+Wnt3a, was incubated with butyl sepharose. Insoluble material was prepared under reducing conditions in 1% SDS immediately before incubation with hydrophobic matrix (see Methods for details), which is likely to disrupt any pre-existing protein interactions. Bound proteins were eluted from the matrix with the mild non-ionic detergent Triton X100 (TX100), or with denaturing SDS. While a significant quantity of Wnt3a alone elutes with TX100, the majority of Oto-modified Wnt3a requires SDS for elution (Fig. 3e). Under these conditions, the Oto protein elutes in a distinct peak from Oto-modified Wnt3a (not shown), indicating that Oto and Wnt3a bound to the matrix independently. These data reveal that Oto-modified Wnt3a is intrinsically more hydrophobic than Wnt3a itself.

**Figure 4:**
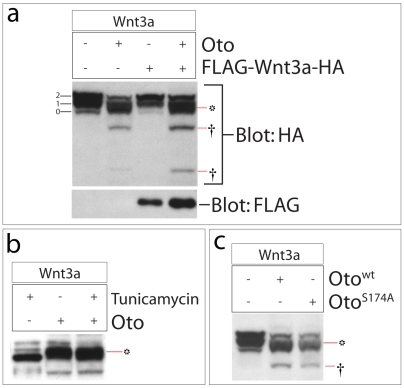
Modification of Wnt3a is independent of signal peptide cleavage, N-glycosylation, and gpi deacylase function. a. The signal peptide of Wnt3a was flag-tagged by cloning Wnt3a into the pFlag7 expression vector (Sigma). The signal peptide is efficiently cleaved from Wnt3a in the absence and presence of Oto. b. Oto robustly modifies Wnt3a in the presence of tunicamycin, an inhibitor of N-glycosylation. c. The gpi deacylase function of Oto was destroyed by mutating the active site Serine (S174) to Alanine. The resulting mutant protein still modifies Wnt3a. Oto-modified Wnt3a bands (*) are indicated throughout; † indicates a degradation product.

Although Oto-modified Wnt3a migrates more slowly than naked Wnt3a, it is not glycosylated (Fig. 3c); its signal peptide has been cleaved (Fig. 4a); and its formation is independent of N-glycosylation (Fig. 4b). The gpi deacylase activity of PGAP1 is supplied by a lipase domain with a serine-dependent active site [49]. Previous work has established that point mutation of the catalytic serine (S174) eliminates deacylase function [49]. We have not tested directly for this gpi deacylase activity in Oto. Based on the high homology to rat PGAP1 and the predicted presence of the same lipase domain in Oto, it is quite likely that Oto also has the deacylase function, but this is not strictly proven. Nevertheless, we mutated the serine residue within the putative active site to an alanine (S174A) in mouse Oto as was done in PGAP1 [49]. S174A Oto still modifies Wnt3a (Fig. 4c), indicating that hydrophobic modification of Wnt3a is a distinct and novel function of the Oto protein. At this time it is not clear whether Oto functions as a catalyst for the Wnt modification reaction, or as a required cofactor.

### Oto-modified Wnt3a is retained in the ER

**Figure 5:**
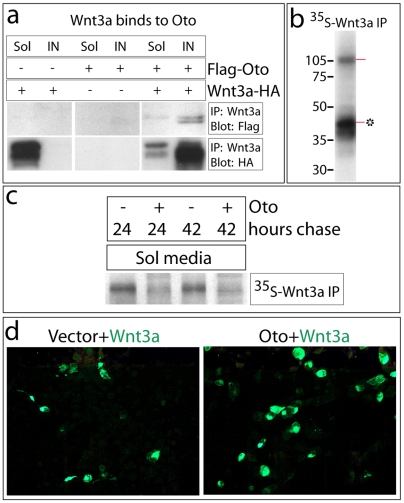
Oto associates with Wnt3a and reduces its secretion. a. Immunopurification of Wnt3a-HA from soluble (Sol) and insoluble (IN) transfected cell extracts using HA matrix. Oto co-immunoprecipitates with Wnt3a in the soluble and insoluble fractions (right), without binding HA matrix (center). b. Immunoprecipitation of Wnt3a from insoluble extracts of metabolically-labeled Oto+Wnt3a cells shows that a protein that is correctly sized to be Oto associates with Wnt3a in the apparent absence of other major binding partners. * = Oto-modified Wnt3a. c. Cells were metabolically labeled, and soluble Wnt3a-HA was HA-immunopurified from conditioned media (Sol media) at the indicated times. Less Wnt3a is recovered from the medium of Oto+Wnt3a cells, indicating that Oto inhibits Wnt3a secretion. d.Wnt3a was visualized by HA-mediated epifluorescence (green) in fields of confluent transfected cells. The Wnt3a signal is much brighter in Oto+Wnt3a cells, reflecting a greater amount of Wnt3a within Oto co-expressing cells.

In order to characterize the interaction between Wnt3a and Oto, full length Wnt3a was immunoprecipitated from transfected cell extracts using an HA affinity matrix. When Wnt3a is immunopurified from soluble extracts of cells co-expressing Oto and Wnt3a under non-denaturing conditions, Oto is also recovered, suggesting that Oto and Wnt3a associate in cells (Fig. 5a). To examine hydrophobic Oto-modified Wnt3a, proteins were extracted from insoluble pellets using denaturing/reducing conditions (see Methods for details). When Wnt3a is immunoprecipitated from such insoluble extracts of Oto+Wnt3a cells, Oto is robustly co-immunoprecipitated, again suggesting that Oto and Wnt3a interact (Fig. 5a). Oto expressed alone does not bind to the HA matrix (Fig. 5a). In a separate experiment, Wnt3a was immunopurified from ^35^S-labeled Oto+Wnt3a insoluble extracts using the same method. The major protein that co-immunoprecipitates with modified Wnt3a is correctly sized to be Oto (Fig. 5b). While it appears that Oto and Wnt3a bind directly, other linking proteins or binding partners may be present at low concentrations, and although we think it unlikely, it is possible that minor residual disulfide bridges and/or protein denaturation may contribute to the observed Oto/Wnt association.

Since Oto is an ER-resident protein like its rat ortholog PGAP1 [49], our observation of an association between Oto and Wnt3a raised the possibility that Oto acts to retain Wnts in the ER. To examine this directly, Wnt3a was immunoprecipitated from the media of ^35^S-labeled cells. Reduced levels of Wnt3a are recovered from Oto+Wnt3a conditioned medium compared to Wnt3a medium, indicating that Oto decreases Wnt3a secretion (Fig. 5c).

When Wnt3a is visualized by HA-mediated immunofluorescence, it localizes predominantly to the ER as previously reported (not shown) [1]. Immunofluorescent images of Wnt3a protein in equivalent fields of confluent transfected cells show an obvious increase in the Wnt3a signal in cells that were co-transfected with Oto as apposed to empty vector, consistent with Wnt3a being cell-retained by Oto (Fig. 5d and data not shown). The greater Wnt3a brightness in Oto cells was seen repeatedly and reproducibly, and is not due to differences in transfection efficiency. Thus four separate experimental results combine to indicate that Oto retains Wnt3a in the ER: Wnt3a's EndoH-sensitive state when co-expressed with Oto, association of the Oto and Wnt3a proteins, reduced Wnt3a secretion in Oto co-expressing cells, and increased Wnt3a signal intensity in Oto/Wnt3a co-transfected cells.

### Oto modifies and ER-retains Wnt1

**Figure 6:**
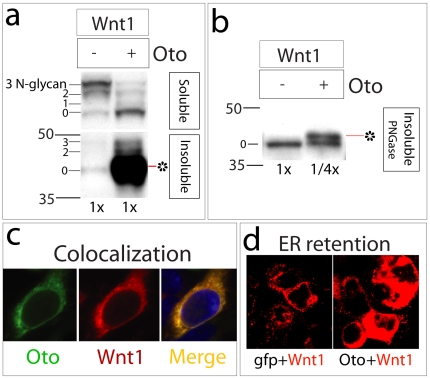
Oto modifies Wnt1 and retains it in the ER. a.Oto modifies Wnt1 (*) robustly when Oto and Wnt1 are co-expressed in 293 cells. 0–3 indicates the number of N-glycans on Wnt1. b. When the quantity of Wnt1 is normalized between control (1×) and Oto (1/4×) samples and N-glycans removed with PNGaseF, it becomes clear that Oto-modified Wnt1 (*) migrates at 42 kDa, like Oto-modified Wnt3a. c. Gfp-Oto (green) and Wnt1-HA (red) were co-expressed and visualized in a single cell using confocal microscopy. They overlap significantly (merge, yellow), indicating that Wnt1 localizes to the ER with Oto. The nucleus is stained with DAPI (blue). d. Wnt1 was visualized by HA-mediated fluorescence (red) in fields of confluent transfected cells. The Wnt1 signal is much stronger in Oto cotransfected cells, reflecting Oto-mediated retention of Wnt1.

To see if Oto modifies other Wnts, we also looked at Wnt1. Like Wnt3a, Wnt1 signals via the canonical pathway and is lipidated by porcupine [7], [12], [13]. Oto and Wnt1 were co-expressed in 293 cells as usual. When equal proportions of extracts are examined, a strikingly large quantity of hydrophobic Wnt1 protein is seen in Oto+Wnt1 cells compared to gfp+Wnt1 (Fig. 6a), much more than is recovered in Oto+Wnt3a cells (Fig. 3b). Co-IP experiments reveal that Oto associates with soluble and insoluble Wnt1 as it does with Wnt3a (not shown). As for Wnt3a, PNGaseF digestion confirms that Oto-modified Wnt1 migrates at 42 kDa, immediately above the naked Wnt1 polypeptide, when Wnt1 quantities are normalized (Fig. 6b). Both soluble and insoluble Wnt1 are recovered from Oto+Wnt1 cells in fully EndoH-sensitive states (not shown), indicating that Oto-modified Wnt1 is located in the ER. To look at protein localization directly, Oto and Wnt1 were immunolabeled and visualized by confocal microscopy in cotransfected cells. The confocal images show that the two proteins colocalize (Fig. 6c). When Wnt1 is immunolocalized in gfp vs. Oto cotransfected cells, the immunolocalized Wnt1 signal is much brighter in Oto co-expressing cells (Fig. 6d). As seen with Wnt3a, co-expression of Oto and Wnt1 is associated with reduced Wnt1 secretion (see panel d of final figure in this manuscript). Therefore the data are consistent with a role for Oto in ER-retaining Wnt1.

### Oto-modified Wnt3a contains a gpi-like anchor

**Figure 7:**
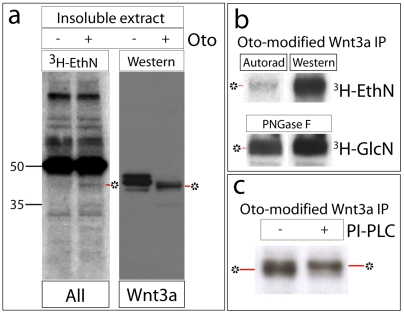
Oto-modified Wnt3a has the hallmarks of a gpi-anchored protein. a. Transfected cells were metabolically labeled with ^3^H-ethanolamine, and all insoluble proteins were resolved by SDS-PAGE, blotted, and exposed to film to visualize ^3^H-ethanolamine-labeled proteins (left). The identical blot was then probed with HA to detect Wnt3a-HA (right). Oto-modified Wnt3a incorporates ^3^H-ethanolamine, while Wnt3a expressed alone does not. b. Transfected cells were metabolically labeled with ^3^H-ethanolamine (upper) or ^3^H-glucosamine (lower) and Wnt3a-HA was immunopurified (IP) from insoluble extracts. Left is autorad, right is Western in both cases. Oto-modified Wnt3a incorporates the gpi anchor components ^3^H-ethanolamine and ^3^H-glucosamine. In the lower panel, immunopurified material was digested with PNGaseF prior to analysis, to remove ^3^H signal due to N-glycosylation. c. Immunopurified Oto-modified Wnt3a was incubated with or without PI-PLC *in vitro*, then analyzed by western blotting. PI-PLC-digested Oto-modified Wnt3a shows a gel upshift (right) compared to undigested (left), due to PI-PLC-mediated cleavage of gpi anchor lipids. * = Oto-modified Wnt3a.

If Oto and Wnt bind directly in the ER under *in vivo* conditions, their association could be sufficient to explain the observed ER retention of Wnts. It is also possible that the dramatic increase in Wnt hydrophobicity independently reduces the Wnt secretion. To explore this question further, we sought additional insight into the nature of the Oto modification. The analysis was continued using Wnt3a, since Oto apparently modifies and retains both Wnts similarly. Because Oto encodes the gpi deacylase PGAP1 [49], we looked for evidence of gpi modification. A gpi anchor is a hydrophobic moiety composed of a conserved core of phosphoethanolamine, mannose (MN) and glucosamine (GlcN) linked via inositol to a doublet of fatty acids (Fig. 2). Gpi anchors are synthesized and covalently attached to proteins in the ER [52]. To see if gpi anchor components are present in Oto-modified Wnt3a, we performed separate metabolic labeling experiments with ^3^H-ethanolamine (^3^H-EthN), ^3^H-GlcN, and ^3^H-MN. When aliquots of all proteins from ^3^H-EthN-labeled insoluble extracts are resolved and blotted, a 42 kDa band consistent with the size of Oto-modified Wnt3a is visible in the autoradiograph (Fig. 7a). Probing the same blot as a Western with HA verifies that the 42 kDa band is indeed Oto-modified Wnt3a (Fig. 7a). This labeling experiment was repeated, except Wnt3a was immunopurified prior to blotting. Again, a likely signal is visible on the ^3^H-EthN autorad, and western blotting confirms that this signal corresponds to Oto-modified Wnt3a (Fig. 7b). The same approach reveals that deglycosylated Oto-modified Wnt3a incorporates ^3^H-GlcN (Fig. 7b) and ^3^H-MN (not shown).

A commonly used method of testing for the presence of a gpi anchor on a protein is *in vitro* cleavage with phosphatidylinositol-specific phospholipase C (PI-PLC; Fig. 2) [53]. PI-PLC is a bacterial enzyme that cleaves lipids off the gpi anchor, and this often results in altered migration of the gpi-linked protein on SDS-PAGE. The prion protein for example shifts upward on SDS-PAGE gels after digestion with PI-PLC [53]. Similarly, following *in vitro* incubation with PI-PLC, we found that immunopurified Oto-modified Wnt3a shows a visible upshift (Fig. 7c).

**Figure 8:**
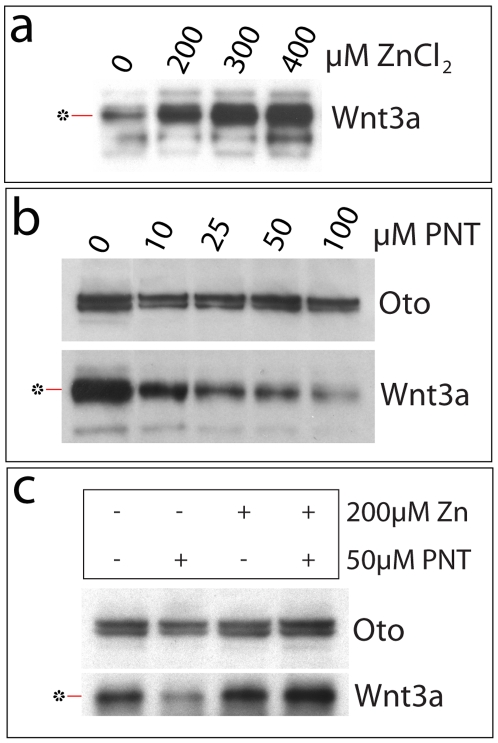
Oto-modified Wnt3a behaves like a gpi anchored protein. a. The addition of ZnCl_2_ increases the yield of Oto-modified Wnt3a (*) in a dose-dependent manner, by stimulating Zn-dependent enzymes of the gpi biosynthetic pathway. b. 1,10-phenanthroline (PNT), a Zn chelator, decreases the amount of Oto-modified Wnt3a by inhibiting gpi biosynthesis, without affecting synthesis of Oto. c. ZnCl_2_ reverses the inhibitory effect of PNT.

Oto-modified Wnt3a behaves like a gpi-linked protein in other fashions as well. For instance, ZnCl_2_ increases gpi anchoring by stimulating Zn-dependent metalloenzymes of the gpi biosynthetic pathway [54], [55]. The addition of ZnCl_2_ to Oto+Wnt3a cells increases the yield of Oto-modified Wnt3a in a dose-dependent manner (Fig. 8a). Conversely, treating cells with the Zn-chelator 1,10-phenanthroline (PNT) inhibits gpi biosynthesis and prevents formation of gpi-anchored proteins [54]. When PNT alone is added to Oto+Wnt3a cells, the quantity of Oto-modified Wnt3a decreases, again in a concentration dependent way, while Oto levels are not affected (Fig. 8b). Co-treating cells with PNT and Zn reverses PNT's inhibitory effect on Oto-modified Wnt3a (Fig. 8c). Additionally, cotransfecting Oto and Wnt3a with PigF, an enzyme necessary for the phosphoethanolamine transferase reaction during gpi biosynthesis [56], increases the yield of Oto-modified Wnt3a (not shown). Collectively, these data demonstrate that the Wnt3a hydrophobic modification exhibits the characteristics of a gpi anchor.

### Wnt activity is premature in oto mutants

**Figure 9:**
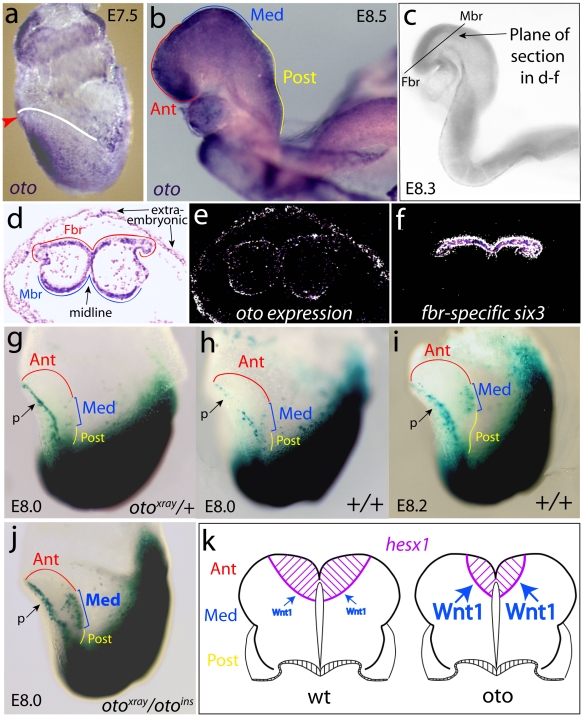
oto deficiency augments Wnt activity in the Wnt1 domain of the early neural plate. *a, b.* Whole mount *in situ* shows that *oto* mRNA (purple) is expressed widely from E7.5–E8.5, correctly placed to act on Wnt1 *in vivo.oto* mRNA is prominent in the anterior of embryos at E7.5 (a, arrowhead = anterior, neural plate outlined in white), and in the neural folds at E8.5 (b, 6–8 somites). c–f. Anterior sections (c) were examined by radioactive *in situ* (e, f). Bright field, labeled view of the section in e (d). Dark field views show *oto* (e), and *six3* (f) in adjacent sections; only expressing cells are visible (white = highest expression level). g–j. Canonical Wnt signaling (blue–green) was visualized in embryos using the BAT-gal Wnt reporter. Embryos are shown in side view, with anterior left; p = presumptive pericardial cells. Neural plate subregions are outlined in distinct colors (Ant = anterior, red; Med = medial, blue; Post = posterior, yellow). *oto^xray^/+* = heterozygote, +/+ = wildtype (wt), *oto^xray/^oto^ins^* = homozygous *oto* mutant. In the neural plates of *oto* heterozygous (g) and wildtype embryos (h), Wnt activity is essentially absent in the medial presumptive Wnt1 domain at E8.0 (1–2 somites). i. In wt embryos, medial neurectodermal Wnt activity first appears at E8.2 (2–3 somites), when the neural plate is larger in size. j. In *oto* mutants at E8.0, robust Wnt signaling is detected prematurely in the medial neurectoderm, when the neural plate is still relatively small. k. A dorso-anterior view cartoon of (posteriorly-truncated) early somite stage neural plates from wt and *oto* embryos with subregions labeled as in g–j, and not showing forebrain defects in the *oto* case. The Wnt activity results in h & j are diagrammed, as are the expression patterns of the Wnt-repressible *hesx1* gene that are typical to each genotype [33]. The arrows represent Wnt1's hypothesized repressive effect on *hesx1*.

Wnt1 is expressed in the medial neural plate in mouse, and is required for the development of the midbrain and anterior hindbrain [57], [58]. The onset of Wnt1 transcription is tightly controlled, with transcripts first detectable in the neural plates of presomite [45], [59] and one somite stage embryos [23], [46], [58], [60], [61]. Wnt1 therefore is expressed in the right time and place to be regulated by Oto, provided that Oto is also expressed in the neural plate. The expression of *oto* mRNA during development was examined from E5.5-18.5 using a combination of northern blots and *in situ* hybridization on whole mount and sectioned embryos. *oto* was expressed at all stages examined. At E7.5, when embryos are gastrulating, *oto* is ubiquitously expressed, showing elevated expression in the anterior midline (Fig. 9a). During E8 when somitogenesis begins, *oto* continues to be expressed throughout the embryo, showing highest expression in the dorsal neural folds (Fig. 9b). To look closely at *oto* expression in the developing forebrain and midbrain during the early neural plate stage (E8.3, ∼4–5 somites), we examined *oto* expression in sections (Fig. 9c–f). In an anterior section that contains forebrain and midbrain regions (Fig. 9c,d), *oto* is ubiquitously expressed (Fig. 9e). In a section immediately adjacent to that in Fig. 9e, the location of the forebrain territory was verified by visualizing expression of the forebrain-specific gene *six3* (Fig. 9f). After E8, *oto* continues to be widely expressed in embryos throughout gestation (not shown). The expression results in Fig. 9a–f are consistent with Oto and Wnt1 being co-expressed in the early neural plate.

Our biochemical studies and the Wnt-like appearance of *oto* defects suggested that altered Wnt signaling is involved in the *oto* phenotype. To examine canonical Wnt signaling in mutant embryos directly, we incorporated the BAT-gal transgenic Wnt reporter, which consists of *lacZ* driven by β-catenin and TCF responsive elements [62]. To maximize chances of seeing defective Wnt signaling, all embryos were minimally fixed and over-stained. Under these conditions, lacZ+ cells were visible within minutes in caudal areas, where Wnt activity is highest.

Sixty-one BAT-gal embryos from *oto* intercrosses ranging from E7.5 to E8.5 were initially examined. In BAT-gal embryos E8.25–E8.5, no differences in neurectodermal or non-neurectodermal Wnt staining were detected (not shown). However, some variation in staining was seen in embryos from E8.0–E8.25. It should be noted that only the anteriormost portions of embryos could be examined from E8.0 to E8.5, because canonical Wnt activity in the posterior portions of embryos is completely stained from E8.0 (Fig. 9g–j). After E8.5, the anterior portions as well are so heavily stained that any differences could not be effectively investigated.

To look at the variations seen in E8.0–E8.25 embryos further, we focussed on a subset of 20 neural plate stage BAT-gal embryos having 0–3 somites. In our analysis, in wildtype underfixed and overstained embryos, Wnt activity in the neural plate is detectable at 2–3 somites at the earliest. In *oto*-heterozygous and wildtype embryos with 1–2 somites (E8.0), no significant Wnt activity is apparent in the anterior neural plate (Fig. 9g,h). In wildtype and *oto* heterozygous embryos (*oto* hets do not show any morphological defects), Wnt activity is first detected in the medial neural plate at 2–3 somites (E8.2, Fig. 9i). At this stage, embryos have visibly larger neural plates than E8.0 embryos, due to the rapid growth occurring during this gestational period. By striking contrast, *oto* mutants show bold staining in the medial neural plate at just 1–2 somites (Fig. 9j), when the neural plate as a whole is still relatively small in size. 3/20 embryos displayed significant staining in the medial neural plate at 1–2 somites, and in each of these staining was localized to the medial neural plate as shown in Fig. 9j. Genotyping of the full set revealed that these 3 embryos were *oto* homozygotes. One additional *oto* homozygote was recovered in the set of 20 that did not show premature Wnt activity. This is not surprising given that the severity of the *oto* phenotype is quite variable; a significant fraction of *oto* homozygotes show no detectable morphological defects [33]. No BAT-gal changes were seen in *oto* hets, which are phenotypically normal [33].

We previously showed that expression of the forebrain-specific and Wnt-responsive *hesx1* gene is reduced in *oto* mutants. Interestingly, we first saw a *hesx1* deficit in mutants at ∼2–3 somites [33]. Current data indicates that canonical Wnt signaling represses *hesx1* expression [30], [44]. Therefore our present and previous results combined suggest that the premature Wnt1 activity in *oto* mutants (Fig. 9j) is responsible for reducing *hesx1* expression in the early *oto*-mutant forebrain; these results are summarized in Fig. 9k.

### Oto knockdown increases Wnt1 secretion and activity

**Figure 10:**
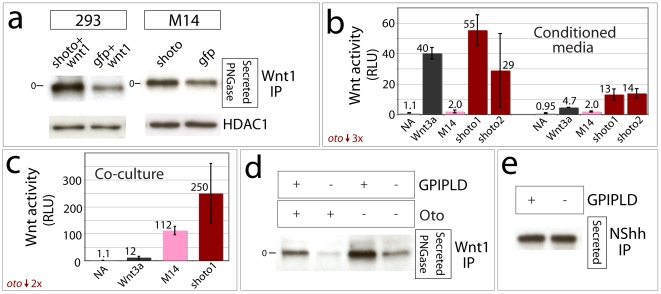
*oto* knockdown increases Wnt1 activity *in vitro*, and GPI-PLD reduces Oto-mediated Wnt1 retention. *a.* Transient shRNA knockdown of endogenous *oto* (shoto) increases the quantity of Wnt1 protein in conditioned media from 293 and M14 melanoma cells. The level of HDAC1 protein in cell extracts does not vary, indicating that cell number remains constant. b. Stable shRNA-mediated knockdown of endogenous *oto* by 3 fold in two independent M14 lines (shoto1 & shoto2) increases endogenous Wnt activity in conditioned media (CM) by at least 6.5 times on average, compared to control CM (M14). Data from two separate experiments are plotted (left, right). CM from experimental cells or purified Wnt3a (50 ng/mL, control) was applied to responding NIH3T3 cells stably expressing a canonical Wnt luciferase reporter. The mean luminescence intensity (RLU) per condition is indicated by number, and each number represents the mean of 10 (left) or 6 (right) independent trials. Error bars represent one standard deviation. c. Stable knockdown of *oto* mRNA by 2 fold in M14 cells (shoto1) augments Wnt activity by an average of 2 fold compared to controls (M14) in a co-culture assay (8 trials per condition). Wnt3a control and data analysis are as described in b. d. Co-expression of gpi-specific phospholipase D (GPI-PLD) and Wnt1 in transfected 293 cells increases secretion of Wnt1 in the presence (left) and absence (right) of cotransfected Oto, suggesting that cleavage of anchor lipids reduces ER retention of Wnt1. e. GPI-PLD does not affect secretion of NShh.

The Oto-mediated ER retention of Wnt1/3a we have observed supports the conclusion that Oto acts to retard Wnt secretion, and conversely suggests that reduced *oto* expression could lead to premature or excessive Wnt secretion. To test the effects of reduced *oto* levels, we designed shRNAs targeting *oto* mRNA in order to knock down endogenous *oto* expression [63]. Two independent shRNAs toward *oto* were designed, which had similar effects. Cotransfection tests illustrated that these shRNAs effectively reduce Oto protein levels (not shown). We used two cell lines in *oto* knockdown experiments, 293 cells and the human melanoma M14 cell line [64]. Neither of these cell lines is canonically Wnt-responsive [26], [65], and so should not show growth stimulation in response to secreted Wnt; as expected, cell numbers remained constant in our knockdown experiments (Fig. 10a and not shown). For 293 cells, Wnt1 was cotransfected with *oto* shRNA (shoto), and for M14 melanoma cells, only shoto was transfected, since these cells express Wnt1 endogenously (data not shown and GNF SymAtlas) [66]. Knockdown of endogenous *oto* in 293 and M14 melanoma cells dramatically increases the amount of soluble Wnt1 recovered from the media (Fig. 10a). As a control, the culture media were examined by immunoprecipitation for the abundant cytoplasmic protein GAPDH, but none was recovered (not shown), revealing that elevated Wnt1 levels are not due to cell lysis. These results indicate that the level of Oto expression correlates with the level of secreted Wnt1; less Oto in the ER results in more Wnt1 in the extracellular space.

In *oto* embryos, we have observed increased Wnt activity in the Wnt1 domain of the neural plate (Fig. 9j). Consistently, in shoto knockdown cells, we have observed increased Wnt1 protein in conditioned media (Fig 10a). However, it is important to determine whether the Wnt1 secreted by *oto* knockdown cells is not only greater in abundance, but also biologically active. To address this, we generated stable shoto knockdown cells from the M14 line. We chose to do activity experiments with M14s because these cells express both *oto* and Wnt1 endogenously. In the first assay, we generated 4-day conditioned media (CM) from shoto and control M14 cells, and applied these to NIH3T3 cells that stably express a canonical Wnt luciferase reporter. NIH3T3 cells are capable of responding to Wnt signals via the canonical Wnt pathway [67]. As a control, purified Wnt3a protein was added to reporter cells at 50 ng/mL. Wnt activity was measured and normalized using a dual luciferase system, and results are expressed as the mean luminescence intensity (RLU) of 6–10 independent trials per condition. The Wnt activity induced by shoto cells consistently exceeded that of control M14 cells by greater than 6.5 fold on average (Fig. 10b). In the shoto1 and shoto2 cell lines, real time RT-PCR revealed that the level of oto mRNA was reduced 2–3 fold relative to control cells (Fig. 10b,c and not shown).

In the CM activity experiments, there was variability in the intensity of the Wnt response (Fig. 10b). Variability between different experimental sets is explained by the age of responding cells; we observed that later passaged NIH3T3 cells responded to CM and purified Wnt3a control protein more weakly than earlier passaged cells (compare Fig. 10b left vs. right). As a separate point, some loss of activity likely resulted from the nature of the Wnt1 protein; Wnts produced endogenously are hydrophobic due to porcupine-mediated lipidations [7], [12], [13] and tend to stick to plastic surfaces in the absence of detergents, causing losses during pipetting, storage, and general handling. To eliminate this potential source of variability, we adopted a co-culture approach, in which Wnt-producing M14 cells were plated together with responding cells. This approach did yield much higher Wnt activity readings than the CM method (Fig. 10c), but did not eliminate variability in the Wnt response elicited by shoto cells. This variability may be an intrinsic property of *oto*-deficient cells, and/or due to the fact that these are knockdown rather than knockout cells. Taken together with the overexpression studies, these activity experiments indicate an inverse dose-response relationship between *oto* expression levels and Wnt activity, with lower *oto* levels resulting in production of more active Wnt signal.

### GPI-PLD relieves retention of Wnt1

A lingering question remains; what is the role of the Wnt gpi anchor itself in Wnt secretion? We addressed this by co-transfecting mammalian gpi-specific phospholipase D (GPI-PLD) with Wnt1 alone or with Oto+Wnt1, and then examined the consequences on Wnt secretion. GPI-PLD is an enzyme that specifically cleaves lipids from gpi anchored proteins, and it can do so within the ER [68]. Consistent with the idea that it is a gpi anchor that modifies Wnt1, co-expression of GPI-PLD dramatically increases the levels of soluble Wnt1 in conditioned media in gfp and Oto co-transfected cells (Fig. 10d). Conversely, GPI-PLD has no effect on secretion of the control protein NShh in the absence (Fig. 10e) or presence (not shown) of co-transfected Oto. These results indicate that intracellular cleavage of gpi anchor lipids relieves ER retention of Wnt1, and in so doing augments its secretion.

## Discussion

We present evidence that Oto/PGAP1 functions as a novel component of the Wnt pathway during mouse forebrain development. Interestingly, another study has recently identified *Ci-PGAP1* as an essential component of the canonical Wnt pathway during embryogeneis of the chordate *Ciona intestinalis* [69]. This study and ours support the idea that Oto/PGAP1 can function both as a gpi deacylase and as a regulator of Wnt signaling. We further show that overexpression of Oto leads to a dramatic increase in the hydrophobicities of co-expressed Wnt3a and Wnt1. Oto modification may extend to other Wnts, given the high degree of protein conservation in this family, and the widespread expression of *oto* in embryos and adults. Our preliminary results indicate that Wnt5a is also Oto-modifiable (not shown). Gpi-anchoring by the widely expressed Oto protein likely contributes to explaining why Wnts are poorly secreted by mammalian cells [1].

The characterizations reported here show that the hydrophobic modifications on Wnt3a and Wnt1 have the hallmarks of gpi anchors. While the appended moiety itself is clearly a chemical relative of the conventional gpi anchor, the process by which the protein is modified is apparently unrelated. For instance, a conventional gpi anchored protein is produced by a transamidation reaction in which the carboxy terminus of the protein is cleaved and replaced with the gpi anchor [52]. In the case of Oto-modified Wnt3a and Wnt1, the carboxy termini remain intact, and the gpi-like anchor is attached internally by an as yet undefined reaction mechanism (unpublished observations). Furthermore, a conventional gpi anchor allows its linked protein to become tethered to the plasma membrane via its anchor lipids, after the protein has exited the secretory pathway [70]. Despite our careful analysis, gpiWnts have not been detected on the plasma membrane. Instead, our immunolocalization and enzymatic analyses indicate that gpiWnt accumulates and lingers in the ER in an immature, hydrophobic state. Further studies are necessary to determine the site of gpi anchorage in Wnts, although these studies are not at all trivial given the extreme hydrophobicities of Wnts 1 and 3a, which are not only gpi-linked, but covalently modified by at least two other lipids as well [5], [12], [13].

Others have shown that porcupine is responsible for adding palmitic acid and palmitoleic acid to Wnt3a at C77 and S209 respectively [5], [12], [13]. Our data in Figure 3 indicate that the Oto modification continues to be added to Wnt3a when C77 is deleted. Our subsequent data show that the Oto modification is a gpi-like anchor. These results demonstrate that the Oto modification does not depend on porcupine's modification of C77, and further that the hydrophobic groups added by porcupine and Oto are distinct in nature. Moreover, these results raise the possibility that the Oto modification proceeds independently of porcupine, although to prove this unequivocally, we would have to mutate S209 also. Alternatively, although Oto and porcupine add different lipid groups to Wnts, it is possible that Oto and porcupine have competitive or synergistic effects during Wnt production.

While the sites of porcupine modification are known, we do not know the sites of gpi anchor addition in Wnts 1 and 3a, and our preliminary work indicates that this is a complex problem. We therefore devised an alternative way to assay the function of the Wnt gpi anchor in Wnt secretion. Rather than preventing anchoring through site specific mutagenesis and then testing the effect on Wnt secretion, we asked the cell to remove the anchor by cotransfecting Oto and Wnt1 with GPI-PLD. If the gpi anchor mediates Wnt retention, then cleaving anchor lipids should release it and allow the protein to proceed through the secretory pathway. The GPI-PLD experiment supports the idea that cleavage of anchor lipids relieves ER retention (Fig. 10d,e). These data in turn suggest that a major role of anchor lipids may be to retain Wnts in the ER via tethering to the ER membrane. Although we have not proved that GPI-PLD cleaves lipids from gpiWnt1 directly, our results are consistent with this conclusion, and are supported by the observation that PI-PLC, the bacterial equivalent of GPI-PLD, cleaves anchor lipids from Oto-modified Wnt3a *in vitro* (Fig. 7c). The detected Oto/Wnt association may contribute to Wnt retention as well. Alternatively or in addition, under Oto-abundant conditions another step may be necessary to release Wnt1 from the ER in a maximal way, such as degradation or inactivation of the Oto protein.

By contrast, our finding that extracellular levels of NShh are not changed by GPI-PLD co-expression suggests that NShh is not modified by gpi (Fig. 10e). Similarly, preliminary Oto/NShh and gfp/NShh co-expression experiments showed no apparent signs of NShh modification (not shown). The lack of evidence for NShh modification thus far suggests that the enhancing effect of GPI-PLD on Wnt1 secretion is due to the specific action of GPI-PLD on gpi-linked proteins, and not to a general effect on all secretory proteins. Additionally, the greatest augmentation in extracellular Wnt1 levels was observed in cells not cotransfected with Oto (i.e., gfp/Wnt1/PLD cells). Importantly, this suggests that Wnt1 is modified endogenously in the 293 cell line. Accordingly, the 42 kDa insoluble Wnt1 isoform was occasionally seen on Western blots of insoluble material from control gfp/Wnt1 transfected 293 cells (not shown). In preliminary studies, we have found evidence for endogenous gpi-anchored Wnt1 in untransfected M14 melanoma cells as well (JSZ unpublished), which express high levels of Wnt1 [66]. Detection of gpiWnt1 in endogenous settings strongly suggests that Oto-dependent gpi anchoring of Wnts has a genuine role in the cell, and is not an artifact of overexpression.

We speculate that Oto-dependent gpi anchoring provides a mechanism for accumulating Wnt proteins as gpi-linked isoforms within Wnt producing cells for release at a later, regulated time point. In such a hypothetical scenario, Oto/Wnt co-expressing cells would generate a supply of Wnt held in reserve in the ER via gpi anchoring. An endogenous gpi-phospholipase would then remove anchor lipids from Wnt to trigger resumption of Wnt maturation, thereby yielding a regulated burst of Wnt ligands. When *oto* is lacking however, our evidence suggests that Wnts travel through the secretory system in a relatively uninhibited manner. In shRNA-mediated *oto*-deficient 293 and M14 cells, significantly more soluble Wnt1 is secreted into the media during a set time compared to controls (Fig. 10a). In M14 *oto* knockdown cells, secreted Wnt1 is also highly active (Fig. 10b,c). These data indicate that Wnt ligands secreted by *oto*-deficient cells are mature, active, and greater in quantity due to reduced Oto-mediated ER retention. We were not able to look directly at ER localization of Wnts in embryos. To our knowledge, antibodies that detect mammalian Wnt proteins at endogenous expression levels have not yet been generated [4].

The *oto* mutant phenotype is consistent with our biochemical studies. In *oto*-deficient embryos, Wnt signaling in the Wnt1 domain of the neural plate is detected earlier than normal, at 1–2 somites. In the context of our biochemical results, the simplest explanation is that active Wnt1 is secreted prematurely in the mutant due to decreased Oto-mediated ER retention. In *oto* mutants, the domain of Wnt activity in the medial neural plate is similar in size and area to that seen in older wildtype embryos (Fig. 9i,j). But importantly, in the mutant the Wnt activity domain is in the context of a neural plate that is smaller overall. Consequently, the area allocated to develop as forebrain is smaller than normal. Therefore the *oto* neural plate resembles an ectopic Wnt situation, in which the medial Wnt domain overlaps the posterior region of the forebrain competence zone. Moreover, at 1–2 somites when we detect premature Wnt1 activity in *oto* mutants, studies show that murine neurectoderm is uncommitted and labile enough to be respecified with regard to fate [47]. Thus the relatively large area of Wnt activity in *oto* neurectoderm is expected to posteriorize the anterior neural plate, resulting in loss of forebrain cells (Fig. 1d–h) [33].

Posteriorization of the *oto* forebrain may occur by two possible mechanisms. First, the Wnt activity visualized in Fig. 9j does not cover the anteriormost neural plate, suggesting that anteriormost cells may not directly experience Wnt signaling in *oto*. In this case, posteriorization of the *oto* forebrain may occur by means of a second signal, a relay mechanism. Or, a Wnt activity gradient may extend all the way to the anterior in *oto*, with the lowest levels of the gradient falling below the level of detection. Although there are precendents for morphogenetic signals being propagated by relay [71], [72], [73], the Wnt gradient mechanism seems more apt. Indeed, evidence indicates that Wnt signaling has a role even in anteriormost development [74]. When *tcf3*-mediated Wnt signaling was blocked during *Xenopus* embryogenesis, expression of extreme anterior markers was inhibited [74]. Given this result and the strong evidence for the existence of a functional Wnt gradient in the vertebrate neural plate [14], [15], [16], [19], [21], [22], [23], [24], it seems more likely that loss of the forebrain in *oto* results from a Wnt gradient that fails to taper to its lowest levels, due to a foreshortened forebrain domain. Direct examination of the indicated Wnt gradient will require the development of more sensitive *in vivo* methods for detecting Wnt ligands and/or Wnt activity.

Wnt1 expression studies by others support the gradient mechanism, and indicate that Wnt1 expression in mouse normally extends into the posterior forebrain domain of the early neural plate. From 1–3 somites, a Wnt1-lacZ reporter shows a soft, unrefined anterior Wnt1 border with lacZ+ cells trailing into the forebrain domain [45], [46]. At four somites, a 5.5 kb Wnt1 enhancer drives lacZ expression in a pattern similar to normal Wnt1, with lacZ cells appearing within the posterior forebrain [59], [60]. At neural tube stages, Wnt1 is expressed in the dorsal neural tube with an anterior limit at the zona limitans intrathalamica (ZLI) [46]. The ZLI is a signaling center that forms within the diencephalic portion of the posterior forebrain [75], [76]. The finding that ZLI positioning is Wnt-regulated in the neural plate in mouse, chick, and zebrafish [20], [22], [77], [78] suggests that Wnt activity normally extends into the prospective posterior diencephalic region at early somite stages. Therefore it is quite possible that a premature, anteriorly shifted Wnt1 activity gradient is solely responsible for posteriorizing the *oto* forebrain. Alternatively, another as yet unidentified Wnt may also be involved, although if so, the activity of this other putative Wnt would be reflected in the BAT-gal readout.

Placing our results in a molecular context, the earliest *oto* forebrain defect was detected at 2–3 somites, when *hesx1* expression is reduced in the anterior neurectoderm [33]. Hesx1 function is required in the anterior neurectoderm itself, not in anterior mesendoderm or anterior visceral endoderm, in order to preserve forebrain development [37]. Recent data reveal that *hesx1* expression is repressed by canonical Wnt signaling [30], [44]. Our results suggest that the observed premature Wnt signaling in *oto* mutants is responsible for repressing *hesx1* in the anterior neural plate. By correlation, *hesx1* and *six3*-deficient embryos show similar forebrain defects to *oto* mutants, and also show excessive Wnt1 expression and/or Wnt activity in the neural plate [23], [30], [36], [37]. In these latter mutants however, the primary defect is loss of the Wnt-repressive effects of Hesx1 or Six3, which then allows derepression of Wnt1 in anterior neural cells. In the *oto* case, our data indicate the reverse, and suggest the primary *oto* defect is prematurely robust Wnt activity in the Wnt1 domain, which then represses *hesx1* by encroaching upon its domain from the posterior direction (Fig. 9k). Our combined biochemical and phenotypic results support the following interpretation of *oto* forebrain defects. Decreased ER retention of Wnt1 in *oto*-deficient neurectoderm leads to premature Wnt signaling in the Wnt1 domain; in the younger, smaller *oto* neural plate, this Wnt activity covers too much of the anterior neural region, repressing *hesx1* expression and curtailing anterior neural development.

While it is possible that dysregulation of other secreted proteins may contribute to *oto* forebrain defects, the salient premature neurectodermal Wnt activity in the *oto* mutant is likely to be the major factor contributing to the forebrain deficit, given the demonstrated potency of Wnts in mediating neural posteriorization in vertebrates [18], [23], [25], [26], [27], [28], [29], [30], [31], [32], [34], [35], [78]. Other candidate proteins that Oto might hypothetically regulate are expected to be limited to the set of secretory proteins involved in patterning the regions affected by *oto* mutation, the head and the vertebrae. Because *oto* is widely expressed in embryos and adult tissues and is essential for survival [33], the *oto* mouse is likely to provide insight into other Wnt-dependent processes in mammalian organisms. Significantly, we have also uncovered an independent role for Oto in regulating development of the hippocampus (unpublished observations). The variety of Wnt-related phenotypes in the *oto* mutant, together with evidence that at least two distinct Wnts can be Oto-regulated, indicate that Oto plays a role in determining the timing or level of Wnt activity in multiple contexts.

In conclusion, the most novel and exciting aspect of the *oto* forebrain phenotype is the indicated underlying molecular mechanism. In other mutant mouse lines referenced here [23], [30], [34], [35], [36], the responsible genes encode Wnt antagonists that repress Wnt transcription or the Wnt response. As a result of these and other studies, much is known about Wnt regulation at the level of the responding cell. By contrast, little is known about Wnt regulation at the level of ligand production [8], [9], [10]. Our results shed new light on the latter process, indicating that Oto regulates Wnt signaling by retaining Wnts in the ER of Wnt producing cells via gpi anchoring. Our results also demonstrate that Oto-mediated Wnt regulation is critical for development of the mammalian anterior forebrain, which includes the telencephalon, the largest and most evolutionarily advanced portion of the mammalian brain.

## Methods

### Ethics Statement

All mice were treated in accordance with the established rules and recommendations of our IACUC protocols and UCSF's animal care committee, and with the welfare of the animals carefully considered.

### Genetics

The *oto^xray^* mutation was successively backcrossed onto C57BL6/J and mapped by standard meiotic mapping. Animals were genotyped using PCR-based, polymorphic microsatellite markers (www.broad.mit.edu/mouse/) that vary by size between C57BL6/J and DBA2/J (Fig. 1a). New markers were designed to binary repeats in the *oto* interval. The MICER insertional allele of *oto* [48] was introduced into ES cells, then into blastocysts. BAC DNAs (http://bacpac.chori.org/) were prepared using the Qiagen Large Construct kit, sizes verified on FIGE gels, and transgenic mice were made. Mice doubly heterozygous for a BAC and the *oto^xray^* mutation were crossed to *oto^xray^* heterozygotes. *oto/oto*; BAC+/− mice were born at normal Mendelian frequency and were viable and fertile (Supplementary Table S1). Mice doubly heterozygous for BAT-gal and an *oto* mutation were crossed to *oto* heterozygotes; resulting embryos were fixed for 5 min in cold 4% PFA, rinsed, stained overnight at room temperature with Xgal, and post-fixed. Stained embryos were individually examined and photographed, then genotyped.

### Molecular Biology & Reagents

A sequence verified, wildtype *oto* cDNA was obtained by RT-PCR from adult mouse kidney, and cloned into the pFlag7 expression vector (Sigma). Mouse Wnt3a and Wnt1 DNA expression constructs encoding Wnts tagged with HA at their carboxy termini were purchased from Upstate. Wnt3aΔ31–107 was made by removing the ScaI/SmaI fragment from mouse Wnt3a. Candidate genes for *oto* were evaluated by RT-PCR, whole mount *in situ* [33], [79] and Northern blotting (Northern Max kit, Ambion). shRNA toward *oto* (AAGAAGCCAAACCATACAAAGTT) was cloned into the pSiRPG vector. Antibodies were purchased from the following sources: mouse anti-flag M5 (Sigma); rat anti-HA high affinity (Roche); goat and rabbit anti-Wnt1 (R&D Systems, Abcam, Zymed/Invitrogen); rabbit anti-HDAC1 (Cell Signaling); secondaries (Amersham, Rockland). Reagents: protein G agarose (Roche), Egfp and dsRedER vectors (Clontech), EndoH and PNGaseF (NEB).

### Cell culture and Biochemistry

293 cells were cultured in DMEM (Invitrogen), and M14 cells in RPMI-1640 (ATCC), each plus 10% fetal calf serum (Hyclone), in 5–6% CO_2_. Transient transfections were done using Cytofectene (BioRad, discontinued) or the equivalent Metafectene (Biontex, Germany). Transfected cells were cultured for 42–48 h, scrape harvested, and lysed in Hepes-buffered RIPA (20 mM Hepes pH 7, 150 mM NaCl, 0.5% sodium deoxycholate, 0.5% NP–40, 0.1% SDS, Roche Complete protease inhibitors). Lysate viscosity was reduced with benzonase nuclease (Novagen). Lysates were spun at maximum speed for 10 min at 4°C, and supers collected in fresh tubes to yield soluble extracts. Insoluble extracts for immunoprecipitation (IP) were prepared by resuspending pelleted material in a small volume of 1% SDS, 20 mM Hepes pH 7, 150 mM NaCl, 10 mM DTT, heating for 10 min at 80°C, diluting 10× with SDS-free RIPA, then spinning at max speed for 10 min to pellet debris, and collecting supers in fresh tubes. Wnts were immunoprecipitated for at least 3 nights. Insoluble extracts for gels only were prepared by solubilizing pelleted material in 0.5% SDS, 1% β-mercaptoethanol. Samples were analyzed by reducing SDS-PAGE on 4–12% or 10% Bis-Tris gels in MOPS (Invitrogen). Proteins were transferred onto PVDF (Amersham and Millipore) using the X-Cell II system (Invitrogen) at 30V for 1.5–2 h on ice. Proteins on blots were detected with ECL Plus (Amersham) and Kodak Biomax Light film. Cells were labeled by adding 140 µCi/ml of 2∶1 ^35^S-methionine∶^35^S-cysteine (ARC) for 30 min, or 0.5 mCi/mL ^3^H-labeled reagent (ARC) for 6–24 h. To visualize ^35^S, gels were dried and exposed to film. For ^3^H, gels were blotted and exposed to film for one month at −80°C using Kodak Transcreen LE intensifying screens. For butyl sepharose (Amersham) chromatography, insoluble extracts were prepared as for IP except they were diluted with RIPA lacking detergents, then incubated with butyl beads for 90 min at room temperature. Beads were washed, then eluted with Hepes-buffered solutions. For PI-PLC digests, 5 µL of immunoprecipitated modified Wnt3a on HA beads was sonicated in 15 µL PBS+0.1% Triton X-100 for one hour, then PI-PLC (Glyko) was added or not, and samples incubated overnight. In *oto* knockdown assays (Fig. 10b,c), shRNAs toward *oto* were transfected, and cells+media harvested after 5 days. For GPI-PLD experiments, gfp or Oto DNA was mixed with NShh DNA 4∶1, and then one part GPIPLD DNA was cotransfected with one part 4∶1 mix. For IPs from media, media was collected, residual cells pelleted and removed, and RIPA reagents, protease inhibitors, and sodium azide added. Media was then precleared with protein G agarose overnight, and the desired protein immunoprecipitated.

### Wnt activity assays

For activity experiments, stable shoto lines in M14 cells were generated by selecting shoto-transfected cells with 1 µg/mL puromycin. Wnt reporter cells were made by stably transfecting NIH3T3 cells with a canonical Wnt luciferase reporter, constructed by cloning the enhancer element of Super8xTOPFlash containing 8 TCF/LEF binding sites into the pGL4.28 vector (Promega) upstream of the minimal promoter element. Luciferase-mediated luminescence was measured using the Dual-Glo kit (Promega). In conditioned media (CM) experiments, cells were plated and CM was collected after 4 days. CM or purified Wnt3a at 50 ng/mL was applied to reporter cells for 6 hours. In co-culture experiments, on day 1 NIH3T3 reporter cells were plated at 20,000 cells per well (24-well plate) and transfected with the Renilla luciferase normalization control vector RL-SV40 (Promega). On day 2, 40,000 M14 or shoto cells were plated on top of the reporter cells. Control reporter cells (no co-culture) were stimulated with 50 ng/mL Wnt3a. On day 3, cells were lysed and luciferase activity measured. In all experiments, each condition was repeated 6–10 times, and the mean luminescence intensity was calculated and plotted with one standard deviation. For measuring *oto* expression in stable shoto cells, mRNA was extracted from one million cells per line in triplicate, and RT-PCR products quantitated using a real-time assay.

## Supporting Information

Table S1 BAC transgenic rescue of *oto*. *otoxray* carriers were intercrossed with animals heterozygous for both *otoxray* and a BAC encoding the genomic *oto* gene. Representative data from one rescuing bac, RPCI23-34H22, is shown. Viability was scored. The *otoxray* mutation is perinatal lethal; only 0.4% of *oto* homozygotes have lived to adulthood (2/446 animals), and these were infertile. The observed column lists the number of viable adult animals recovered with the indicated genotypes. Predictions are based on the total number of animals scored. Bac transgenic mutant animals are born at normal Mendelian frequency (yellow), have normal head morphology, and are fertile, revealing that BAC-mediated restoration of *oto* expression eliminates the *oto* phenotype.
(0.35 MB TIF)

Figure S1 Derivation of the MICER insertional allele of *oto*. Homologous recombination of MICER clone MHPN237e12 with the endogenous *oto* locus in the vicinity of exons 5 and 6 results in a duplication and frameshift after exon 6. 0, +1, and +2 show the reading frame between indicated exons. The *otoins* allele is a likely null.
(0.31 MB TIF)
