# Supplementary figures and images for: Wnt Signaling Is Regulated by Endoplasmic Reticulum Retention
Source: PLoS One. 2009 Jul 10;4(7):e6191. doi: 10.1371/journal.pone.0006191 (PMC2703784; doi:10.1371/journal.pone.0006191)

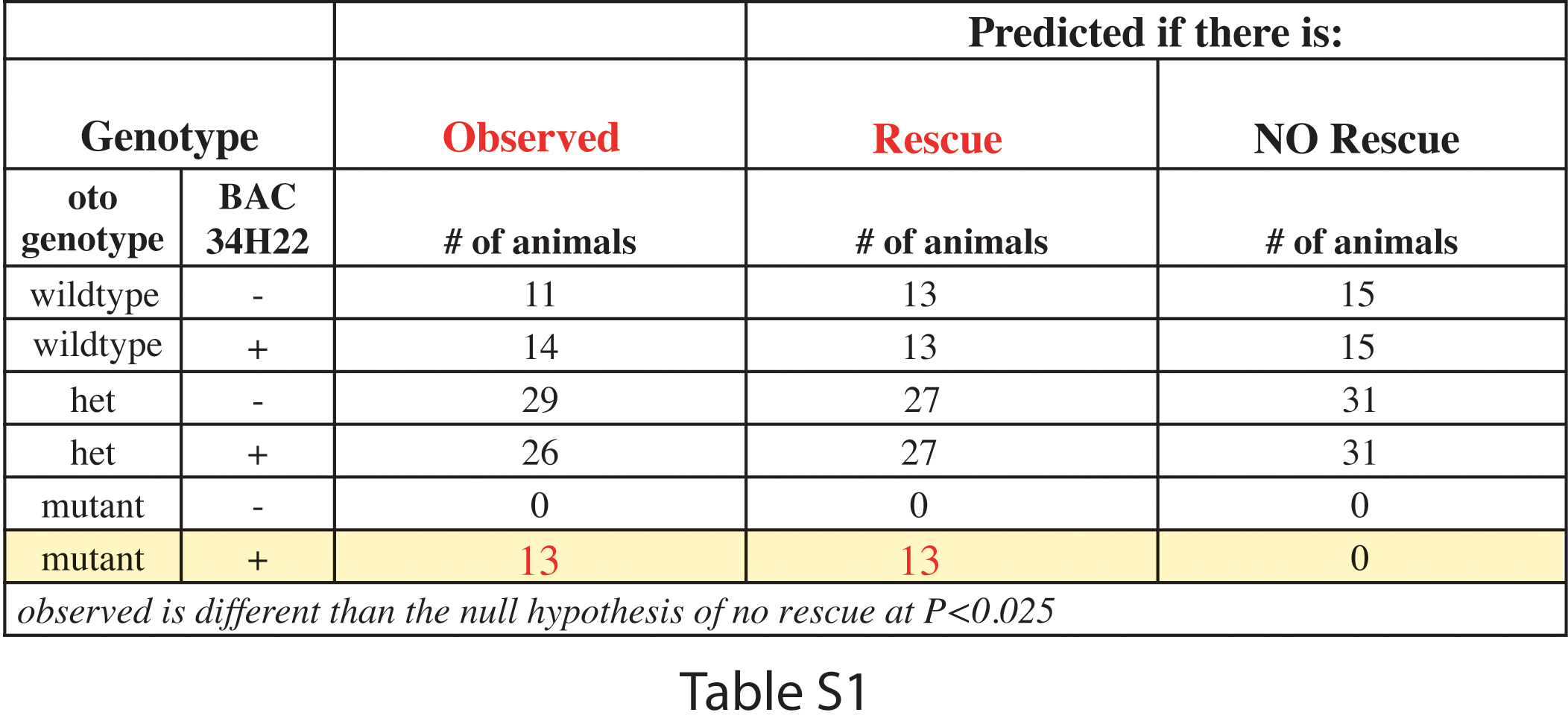

Supplement: Table S1 — BAC transgenic rescue of oto. otoxray carriers were intercrossed with animals heterozygous for both otoxray and a BAC encoding the genomic oto gene. Representative data from one rescuing bac, RPCI23-34H22, is shown. Viability was scored. The otoxray mutation is perinatal lethal; only 0.4% of oto homozygotes have lived to adulthood (2/446 animals), and these were infertile. The observed column lists the number of viable adult animals recovered with the indicated genotypes. Predictions are based on the total number of animals scored. Bac transgenic mutant animals are born at normal Mendelian frequency (yellow), have normal head morphology, and are fertile, revealing that BAC-mediated restoration of oto expression eliminates the oto phenotype. (0.35 MB TIF) [file pone.0006191.s001.tif]

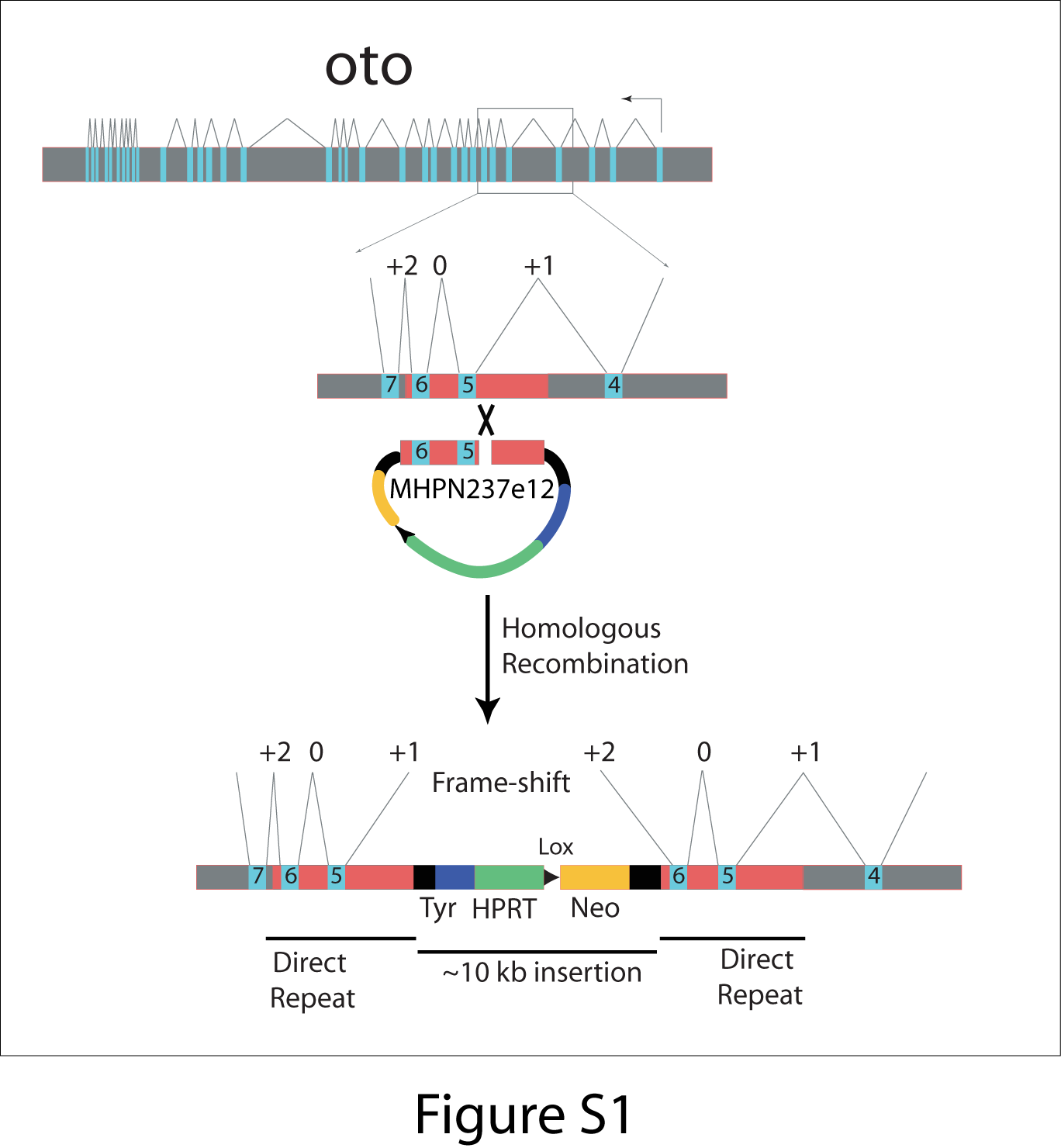

Supplement: Figure S1 — Derivation of the MICER insertional allele of oto. Homologous recombination of MICER clone MHPN237e12 with the endogenous oto locus in the vicinity of exons 5 and 6 results in a duplication and frameshift after exon 6. 0, +1, and +2 show the reading frame between indicated exons. The otoins allele is a likely null. (0.31 MB TIF) [file pone.0006191.s002.tif]
